# The effect of periodontal therapy on the improvement of glycemic control in patients with type 2 diabetes mellitus: A randomized controlled clinical trial

**DOI:** 10.4103/0973-3930.43097

**Published:** 2008

**Authors:** Sukhdeep Singh, Veerendra Kumar, Sheela Kumar, Anitha Subbappa

**Affiliations:** Department of Periodontics, JSS Dental College and Hospital, SS Nagar, Bannimantap, Mysore - 570 015, Karnataka, India

**Keywords:** Glycemic control, glycated hemoglobin, periodontal treatment, type 2 diabetes mellitus

## Abstract

**AIMS:**

The purpose of this study is to investigate the effect of improved periodontal health on glycemic control in type 2 diabetes mellitus (type 2 DM) patients who have generalized periodontitis.

**MATERIALS AND METHODS:**

A total of 45 type 2 DM patients with generalized periodontitis were selected for the study. The selected patients were randomly assigned to three groups (groups A, B, and C) comprising 15 patients each: • Group A received treatment with scaling and root planing only. • Group B received treatment with scaling and root planing followed by systemic doxycycline. • Group C received no treatment (control group). The periodontal parameters recorded included plaque index, gingival index, probing pocket depth, and clinical attachment level. These parameters were recorded at baseline (day zero), at 1 month, and at the end of 3 months. The following metabolic parameters were recorded: fasting blood glucose (FBG), postprandial blood glucose (PPBG), and glycated hemoglobin. These were recorded at baseline (day zero) and at the end of 3 months.

**STATISTICAL ANALYSIS:**

All the parameters were subjected to repeated-measures ANOVA and Scheffe's post hoc test.

**RESULTS:**

A statistically significant effect could be demonstrated for periodontal parameters for both group A and group B (treatment groups). Glycated hemoglobin values showed statistically significant decrease in treatment groups compared to the control group, with group B showing more significant decrease than group A.

**CONCLUSIONS:**

The results of this study showed that nonsurgical periodontal treatment is associated with improved glycemic control in type 2 DM patients.

## Introduction

Diabetes mellitus (DM) and periodontitis are chronic diseases and result from various etiologic factors.[1] A low-grade inflammatory response precedes the development of diabetes mellitus, indicating that diabetes and periodontal disease may be linked via dysregulated inflammatory and immune responses.[2] Patients with DM who have severe periodontitis have impaired glucose tolerance compared to those without periodontitis or with mild periodontitis.[3,4] Periodontal disease is now designated as the ‘sixth complication of diabetes’.[5] The purpose of this study is to investigate the effect of improved periodontal health on the glycemic control in type 2 DM patients with generalized periodontitis.

## Materials and Methods

This study was conducted in the Department of Periodontics, JSS Dental College and Hospital, Mysore. Forty-five patients who visited the Department of Periodontics, JSS Dental College, Mysore, were recruited for this study. All those patients diagnosed with moderate to severe periodontitis and who willingly agreed to participate in the study were considered for inclusion in the study.

### Inclusion criteria

- Patients aged above 30 years of either sex, with type 2 DM
- Absence of any major diabetic complications
- Moderate to advanced periodontitis (30% or more of the teeth examined having ≥ 4 mm probing depth)[6]
- No evidence of any systemic disease other than diabetes being a risk factor for periodontitis.

### Exclusion criteria:

- Patients with uncontrolled DM
- Patients who have undergone periodontal treatment in the 6 month period prior to the study
- History of antibiotic administration within the last 3 months
- Less than 16 remaining natural teeth

### Clinical trial design

Patients were explained the procedure in detail and were included for the study with their consent. Patients were informed that they could withdraw from the study at any time and for any reason. The study protocol was approved by the college ethical committee for the use of human subjects in clinical experimentation.

### Method of collection of data

- In the selected patients, detailed medical history was recorded. The treating physician's consent and details of the patients' diabetes control were also obtained. No change in the medication or diet was made for the patients. None of the patients received any additional guidance for managing their diabetic status.
- The selected patients were randomly assigned to three groups (groups A, B, and C) comprising 15 patients each:
  Group A received treatment with *full mouth scaling and root planing only*.
  Group B received treatment with *full mouth scaling and root planing followed by systemic doxycycline* (100 mg daily for 14 days).
  Group C received *no treatment* (control group).
- After oral examination the teeth with poor prognosis were extracted.
- Patients were instructed to report to the Department of Periodontics after overnight (for 8 h) fasting. Under aseptic conditions 4 ml of venous blood was drawn from the antecubital fossa using vacutainers (BD Vacutainer)™.
- Patients were then given a glucose load, ie, 75 g of anhydrous glucose dissolved in water, and a blood sample was drawn after 2 h.
- The collected blood samples were transported to the pathology laboratory for estimation of the following *metabolic parameters* of the study:
  Fasting blood glucose (FBG)
  Postprandial blood glucose (PPBG)
  Glycated hemoglobin (HbA1C) by liquid chromatography method
- These were recorded at baseline (day zero) and at the end of 3 months.
- For the periodontal status of the three groups, the following parameters were recorded after collection of blood samples:
  Plaque index according to Silness and Loe[7]
  Gingival index according to Loe and Silness[8]
  Probing pocket depth[3]
  Clinical attachment level[3]

**Figure 1:**
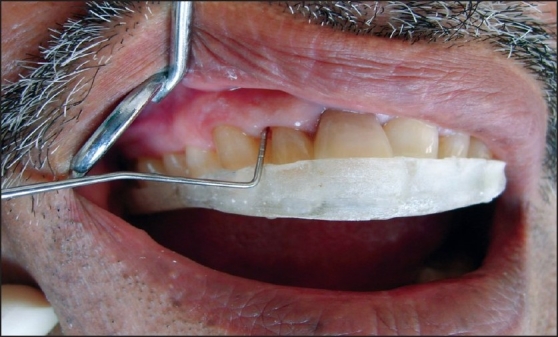
Measurement of the periodontal parameters using occlusal stent.

These parameters were recorded on the remaining teeth. *The parameters were recorded at baseline (day zero), at 1 month, and at the end of 3 months.* Probing pocket depth and clinical attachment level were measured with a William's periodontal probe using customized occlusal acrylic stents [Figure 1].

**Figure 2:**
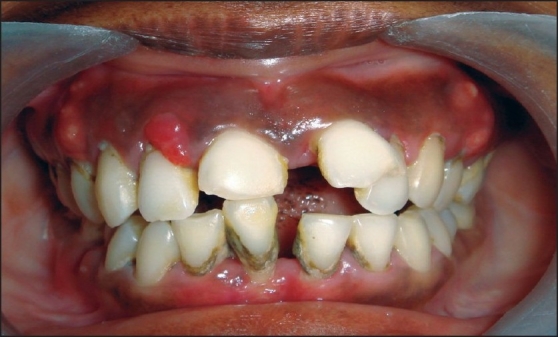
Periodontal condition of a type 2 DM patient with uncontrolled diabetes.

**Figure 3:**
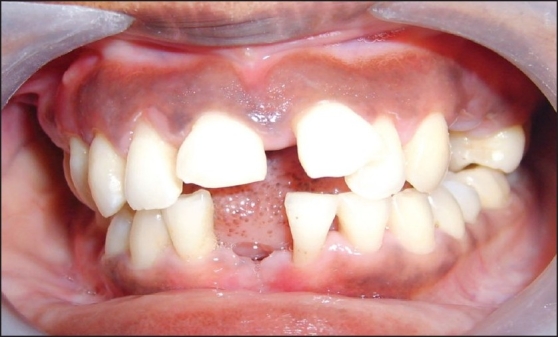
Same patient 3 months after extraction of hopeless teeth and periodontal treatment.
- Group C (control group) received no oral hygiene instructions and full mouth scaling and root planing was not performed in these patients.
- All patients were recalled for periodontal examination at 1 month and at the end of 3 months.
- The blood samples were once again collected at the end of 3 months for measuring the metabolic parameters.

- After recording the periodontal status, patients in group A received oral hygiene instructions and then underwent full mouth scaling and root planing under local anesthesia.
- Patients in group B also received oral hygiene instructions and underwent full mouth scaling and root planing under local anesthesia but, in addition, group B patients were also given doxycycline 100 mg (DOXY-1™, USV Pharmaceuticals), two tablets to be taken on the first day, followed by one tablet daily for 14 days [Figures 2 and 3].

### Statistical analysis

All the parameters were subjected to repeated-measures ANOVA and Scheffe's post hoc test. All the statistical calculations were done using SPSS (Statistical package for the social sciences) for Windows.

## Results

The study group comprised 45 patients. They were randomly divided into three groups of 15 patients each. Group A and group B were the treatment groups, receiving oral hygiene instructions and undergoing full mouth scaling and root planing. Group B, in addition, received 100 mg doxycycline daily for 14 days from the day of commencement of the treatment. Group C was the control group, wherein patients received neither oral hygiene instructions nor full mouth scaling and root planing.

### Periodontal parameters [Table 1] Plaque index

**Table 1:** Clinical periodontal status in treatment and control groups

	Plaque index (mean ± SD)	Gingival index (mean ± SD)	Probing pocket depth (mean ± SD)	Clinical attachment level (mean ± SD)
Group A				
Mean value at baseline	1.72 ± 0.26	1.54 ± 0.30	2.67 ± 0.35	3.44 ± 0.45
Mean value at 3 months	0.83 ± 0.15	0.80 ± 0.22	2.33 ± 0.35	3.14 ± 0.45
Mean difference	−0.89	−0.74	−0.34	−0.30
Statistically significant change at P value = 0.000				
Group B				
Mean value at baseline	1.74 ± 0.15	1.68 ± 0.16	2.52 ± 0.47	3.22 ± 0.63
Mean value at 3 months	0.86 ± 0.12	0.94 ± 0.12	2.14 ± 0.46	2.88 ± 0.61
Mean difference	-0.88	-0.74	−0.38	−0.34
Statistically significant change at P value = 0.000				
Group C				
Mean value at baseline	1.54 ± 0.33	1.64 ± 0.27	2.44 ± 0.26	2.78 ± 0.33
Mean value at 3 months	1.52 ± 0.30	1.64 ± 0.28	2.40 ± 0.46	2.83 ± 0.35
Mean difference	−0.02	0.006	−0.04	0.04
Nonsignificant change				

Following the treatment, there was a mean difference of 0.8933 in the plaque index in group A from baseline to the end of 3 months, while the mean difference for group B was 0.8867 from baseline to the end of 3 months. The results show that group A and group B were comparable in respect to the decrease in plaque scores.

### Gingival index

Both group A and group B showed a mean difference of 0.7400 when evaluated from baseline to the end of 3 months. Again the results showed a similar decrease in the gingival index scores for both the treatment groups.

### Probing pocket depth

The mean difference seen in group B was 0.3800 at end of the 3 months' observation period. This was more compared to the mean difference of 0.3400 seen in group A. The decrease in probing depth in group B was not statistically significant when compared to group A.

### Clinical attachment levels

The clinical attachment gain was more in group B, in which there was a mean difference of 0.3460 at the end of 3 months, whereas group A showed a mean difference of 0.3000 in clinical attachment gain. The greater gain seen in group B was not statistically significant.

Group C (control group) showed no significant changes in any of the periodontal parameters.

### Metabolic parameters [Table 2] Fasting blood glucose

**Table 2:** Metabolic data for treatment and control groups

	Fasting plasma glucose levels (mg/dl, mean ± SD)	2-h postprandial glucose (mg/dl, mean ± SD)	Glycated hemoglobin (mg/dl, mean ± SD)
Group A			
Mean value at baseline	151.6 ± 14.4	190.0 ± 12.4	7.9 ± 0.7
Mean value at 3 months	147.6 ± 14.5	173.4 ± 8.8	7.3 ± 0.6
Mean difference	−3.9	−16.6	− 0.6
	Statistically nonsignificant	Statistically significant difference	
Group B			
Mean value at baseline	164.2 ± 11.7	197.6 ± 12.04	8.3 ± 0.7
Mean value at 3 months	160.04 ± 11.4	175.8 ± 8.3	7.5 ± 0.6
Mean difference	−4.2	−21.8	−0.7
	Statistically nonsignificant	Statistically significant difference	
Group C			
Mean value at baseline	183.4 ± 12.5	205.8 ± 16.9	8.08 ± 0.7
Mean value at 3 months	185.3 ± 12.2	207.5 ± 17.21	8.1 ± 0.74
Mean difference	1.8	1.7	0.06
	Statistically nonsignificant		

Group A showed a mean difference of 3.9 mg/dl from baseline to end of 3 months. Group B showed a decrease of 4.2 mg/dl in mean fasting blood glucose values. The greater decrease in fasting blood glucose levels in group B compared to group A, however, was not statistically significant at a *P* value of 0.05.

### Two-hour postprandial blood glucose

The decrease in mean values for 2-hour post prandial blood glucose was 16.6 mg/dl for group A and up to 21.820 mg/dl in group B. This greater decrease seen in group B was not statistically significant at a *P* value of 0.05.

### Glycated hemoglobin

Group A showed a mean difference of 0.60 percent in glycated hemoglobin values, whereas group B showed a decrease in mean difference of 0.78 percent over the period of 3 months. This change when applied to Scheffe's post hoc test was statistically significant at a *P* value of 0.05 [Tables 3 and 4].

**Table 3:** Scheffe's post hoc test for glycated hemoglobin

Group	n	Subset for alpha = 0.05		
		
		1	2	3
C	15	−0.06		
A	15		0.60	
B	15			0.78
Sig		1.00	1.00	1.00

**Table 4:** Repeated-measures ANOVA for glycated hemoglobin scores

Source	Type III sum of squares	df	Mean square	F	Sig
Change	4.35	1	4.35	474.78	0.00
Change group	3.01	2	1.50	164.51	0.00
Error / change	0.38	42	0.009		

## Discussion

The association between periodontal disease and diabetes has been explored by many researchers over the years.[9–13] It is generally accepted that periodontal disease is more prevalent and more severe in persons with diabetes than in nondiabetic persons. Periodontal signs and symptoms are now recognized as the ‘sixth complication’ of diabetes.[5]

The incidence of periodontitis increases among diabetic subjects after puberty and as the patient population ages.[14] Periodontal disease may be more frequent and severe in diabetic individuals with more advanced systemic complications.[14] The increased susceptibility does not correlate with increased levels of plaque and calculus.[13]

The general signs and symptoms of DM are the direct result of hyperglycemia, and systemic complications of DM are associated with prolonged hyperglycemia. Thus blood glucose level plays a key role in the complications associated with diabetes.[15] Collectively the evidence supports the theory that there is a relationship between the two diseases, especially in patients with poorly controlled DM or hyperglycemia.[14]

The shared susceptibility between diabetes and periodontitis show that both the conditions are linked via mechanisms such as increase in inflammatory cytokines, adipokines, advanced glycated end products, and abnormal neutrophils. The concept of diabetes as an inflammatory state further strengthens the hypothesis that both the diseases are linked via dysregulated inflammatory and immune response.[16–18] There is substantial evidence to support diabetes as a risk factor for poor periodontal health;[2] there is also evidence that periodontal infection adversely affects glycemic control in diabetes. These studies lead to the hypothesis that successful management of periodontal infection will lead to a reduction of the local symptoms of the disease and better control of glucose metabolism.[1,19,20]

More direct evidence regarding the effects of periodontal infection on glycemic control in diabetes comes from treatment studies. There is evidence to support periodontal infection having adverse effect on glycemic control.[1,19,21] However, not all investigators have reported improvement in glycemic control after periodontal therapy.[22,23]

Type 1 diabetes is presumed to originate from an autoimmune process[19] and is usually controlled by the administration of insulin. Glucose levels in these patients are very tightly monitored. Thus, any obvious change in HbA1C might not be evident, although it is possible that insulin requirements might have been lower. Further, patients with type 1 diabetes, in general, may be too young to develop moderate or severe periodontitis. Thus, this study is limited to type 2 DM patients on oral hypoglycemic agents or diet regimen only.[20]

The results of this study show that, following periodontal therapy, there is a statistically significant improvement in glycemic control in individuals with type 2 DM when compared with a nontreatment control group. At baseline, metabolic-matched diabetic patients showed similar levels of plaque accumulation, gingival inflammation, and periodontal breakdown.

Ryan *et al.* reported a decrease in the level of glycated hemoglobin and collagen degradation in diabetic rats following administration of doxycycline or chemically-modified tetracycline. The authors hypothesized that extracellular glycation of proteins in diabetes is inhibited by tetracycline via a non-anticollagenase mechanism. Gingival crevicular fluid and salivary collagenases were also significantly inhibited following administration of systemic tetracycline in labile diabetics as well as in individuals with rheumatoid arthritis. Tetracyclines and their non-antimicrobial chemically-modified derivatives can 1) prevent oxidative activation of latent promatrix metalloproteinases, 2) downregulate matrix metalloproteinases expression and 3) protect the body's major serine proteinase inhibitor (elastase) from both oxidative and matrix metalloproteinase–dependent inactivation. Furthermore, protein synthesis and secretion by periodontal ligament fibroblasts was increased in diabetic rats following tetracycline administration. Overall, this evidence has provided the basis for a therapeutic approach to controlling periodontal disease in individuals with diabetes using tetracyclines and their derivatives.[20,21,24]

Local doxycycline treatment targeted to periodontal tissue appeared to be most effective in reducing blood HbA1C levels.[21] It was postulated that local delivery of doxycycline would be associated with fewer adverse events such as the mild to severe gastrointestinal irritation seen with systemic doxycycline. One of the treatments groups in our study received systemic doxycycline 100 mg/day for 14 days. This was based on the study by Grossi *et al.*[24] None of the patients in our study experienced any adverse side effects with doxycycline.

Our study group comprised 45 patients. They were randomly divided into three groups of 15 patients each. Group A and group B were the treatment groups. Both groups received oral hygiene instructions and underwent full mouth scaling and root planing and, in addition, group B received 100 mg doxycycline daily for 14 days from the day of commencement of the treatment. Group C was the control group, wherein patients did not receive any oral hygiene instructions or periodontal treatment. The periodontal parameters recorded were, plaque index, gingival index, probing pocket depth, and clinical attachment levels. These were recorded at baseline (day zero), at the end of 1 month, and at the end of 3 months. The metabolic parameters recorded consisted of fasting blood glucose, 2-h postprandial blood glucose, and glycated hemoglobin levels. These parameters were recorded at baseline (day zero) and at the end of 3 months.

The result of therapy was assessed after 1 month and at the end of 3 months. There are contradictory opinions in the literature concerning the appropriate time for assessing the healing response to nonsurgical periodontal therapy. Morrison *et al.* and Lowenguth and Greenstein suggested a period of 1 month.[1,19] Badersten *et al.* found that in periodontal pockets of 4–7 mm depth most changes occur in the first 4–5 months, while in deep pockets up to 12 mm, a gradual improvement takes place over a period of 12 months.[1] In this study, the response was evaluated after 3 months as the majority of patients showed a mean probing pocket depths around 3 mm. Patients were recalled and assessed at the end of 1 month to evaluate the response to periodontal therapy and oral hygiene maintenance. In our study, the majority of the patients showed satisfactory oral hygiene maintenance after receiving the oral hygiene instructions. No change in the oral hygiene maintenance was seen in the control group.

The results of this study show that the decrease in plaque index and gingival index were comparable in the two treatment groups. As expected, there was no improvement seen in the control group. The mean probing pocket depth and clinical attachment levels also improved significantly in the treatment groups (group A and group B) compared to control group (group C). The good response of diabetics to nonsurgical therapy in the present study confirms the results of previous investigations.[25]

The results of this study suggest that following periodontal therapy there is a statistically significant improvement in glycemic control in individuals with type 2 DM when compared with a control group. At baseline, metabolic-matched diabetic patients showed similar levels of plaque accumulation, gingival inflammation, and periodontal breakdown. Fasting plasma glucose and 2-h postload plasma glucose are considered important tests for the diagnosis of diabetes. In a patient with diagnosed diabetes, the HbA1C level is used to monitor the patient's overall glycemic control. HbA1C reflects the mean glucose level over the preceding 2–3 months. Thus, the intervals between two consecutive HbA1C tests should be at least 2 months if any relevant changes are to be observed.[15]

There was a decrease in both fasting blood glucose and 2-h postprandial blood glucose levels in our treatment groups compared to control groups but this decrease was not significant. The significant finding of this study is the improvement in the glycated hemoglobin levels seen in the treatment groups. A more important finding is the significant change in glycated hemoglobin levels seen in group B (which received periodontal therapy and systemic doxycycline) compared to group A (which received only periodontal therapy). This finding is in agreement with the studies by Grossi *et al.*, Miller *et al.*, and Iwamoto *et al.*[21,24]

In contrast, previous studies involving periodontal treatment alone reported improvement in periodontal status only. Certain studies, like that done by Stewart *et al.*, reported a decrease in the levels of HbA1C following nonsurgical therapy of periodontitis in type 2 DM patients.[19] They also showed improvement in HbA1C levels in the control group. The authors suggest that this was possibly due to change in diabetic control in some patients. For this reason, in our present study, we did not attempt to change the diabetic control of our patients by giving any additional instructions for control of blood glucose levels.

In an another study by Kiran *et al.*,[1] there was statistically significant difference seen in HbA1C levels in the treatment group which received only scaling and root planing. Here the controls were well matched and they did not show any improvement in metabolic control. The study only included patients with moderate periodontitis and the authors concluded that the improvement in HbA1C levels were due to the improvement in the gingival condition.

Our study incorporates two treatment groups to compare the effect of systemic doxycycline plus scaling and root planing to scaling and root planing alone. The results of this study clearly show that patients in group B, which received adjunctive antimicrobial therapy, showed a better improvement in periodontal and metabolic parameters. A study by Iwamoto *et al.*[21] concluded that antimicrobial periodontal therapy reduces circulating TNF-α, which subsequently reduces circulating insulin concentration and HbA1C level. They also stated that continuous infusion of bacterial lipopolysaccharides and/or TNF-α induces severe insulin resistance in a rat model. Thus, periodontal disease may exacerbate insulin resistance in diabetic patients.

The objectives of the present study were to investigate the effect of improved periodontal health on the glycemic control in type 2 diabetes mellitus patients with generalized periodontitis and to compare the effect of scaling and root planing combined with systemic doxycycline to scaling and root planing alone on glycemic control. The results obtained appear to demonstrate a strong, statistically significant, association between clinical improvement in the periodontal condition and improved metabolic control of diabetes. Moreover adjunctive doxycycline improves the periodontal and metabolic parameters to a statistically significant extent when compared to only periodontal therapy.
